# Supplementary material for: Detection Methods for Aflatoxin M1 in Dairy Products
Source: Microorganisms. 2020 Feb 12;8(2):246. doi: 10.3390/microorganisms8020246 (PMC7074771; doi:10.3390/microorganisms8020246)
Supplement: Supplementary file 1 [file microorganisms-08-00246-s001.pdf]

Table S1. Determination of AFM1 in milk and dairy products using different methods.

Product	Sample Preparation	Extraction	Clean-up	Quantification Method	References
**Milk**	Centrifugation Filtration	Magnetic Solid Phase Extraction (MSPE)	NA[2]	HPLC-FLD	[1]
**Milk**	Centrifugation Filtration	AOAC 2000.08<br>Pepsin-HCl (P-HCl) method	IAC	HPLC-FLD	[2]
**Milk**	Homogenization Centrifugation	C18-SPE columns	NA[2]	HPLC-FLD	[3]
**Milk**	Centrifugation Filtration	NA[2]	NA[2]	Chemiluminescent Enzyme-linked immunosorbent Assay (CL-ELISA)	[4]
**Milk**	Centrifugation Filtration	NA[2]	NA[2]	Fluorometric ELISA	[5]
**Milk**	Centrifugation	NA[2]	NA[2]	Potentiometric aflatoxin M1-immunosensor	[6]
**Milk**	Homogenization Centrifugation Filtration	QuEChERS	QuEChERS C18 sorbent; PSA	UHPLC–MS/MS	[7]
**Milk**	Centrifugation Filtration	NA[2]	NA[2]	Intramolecular electro-chemiluminescence resonance energy transfer (ECL-RET)	[8]
**Milk**	NI[1]	Captiva ND lipids extraction (lipids and proteins removal)- Organic solvents: methanol	NA[2]	Impedimetric aptasensor	[9]
**Milk**	NI[1]	Addition to Apt GMAPs-GO-LAgNPs; Magnetic separation	NA[2]	Electrochemiluminescence (ECL) aptasensor	[10]
**Milk**<br>**Milk powder**	Homogenization	Organic Solvents: Acetonitrile SPE: Oasis HLB cartridge	NA[2]	UHPLC–ESI–MS/MS	[11]
**Milk**	Centrifugation	NA[2]	IAC	HPLC-FLD	[12]

Table S2. (Continued).

Product	Sample Preparation	Extraction	Clean-up	Quantification Method	References
**Milk**	Centrifugation	NA[2]	IAC	HPLC-FLD	[12]
**Milk powder**	NI[1]	Organic Solvents: chloroform<br>Magnetic molecularly imprinted polymer l (MMIP-SB)	NA[2]	HPLC–MS/MS	[13]
**Milk powder**	NI[1]	Organic Solvents: methanol/water	NA[2]	RT-qPCR based aptasensor	[14]
**Milk**	Homogenization<br>Centrifugation<br>Filtration	NA[2]	IAC	HPLC-FLD	[15]
**Milk**	Homogenization<br>Centrifugation<br>Filtration	NA[2]	IAC	HPLC-FLD, TLC, ELISA	[16]
**Milk**	Centrifugation<br>Filtration	NA[2]	IAC	HPLC-FLD	[17]
**Milk**	Centrifugation<br>Filtration	SPE column	NA[2]	UHPLC–QqQ–MS/MS	[18]
**Milk**	NI[1]	Organic Solvents: acetonitrile<br>Organic Solvents: acetone<br>QuEChERS	NA[2]	UHPLC–QqQ–MS/MS	[18]
**Milk**	NI[1]	MIP	NA[2]	Scanning electron microscopy, energy dispersive spectroscopy, and Fourier-transform infrared spectroscopy	[19]
**Milk**	NA[2]	QuEChERS	QuEChERS - C18 sorbent and PSA	UHPLC-Q-Orbitrap HRMS	[20]
**Milk**	Centrifugation<br>Filtration	NA[2]	NA[2]	ELISA, HPLC-FLD	[21]

Table S3. (Continued).

Product	Sample Preparation	Extraction	Clean-up	Quantification Method	References
**Milk**	Homogenization<br>Centrifugation<br>Filtration	NA[2]	IAC	HPLC-FLD	[22]
**Milk**	NI[1]	liquid–liquid extraction - Homogenization<br>Organic Solvents: ethyl acetate, acetic acid	NA[2]	HPLC-UV	[23]
**Milk**	NA[2]	Organic Solvents: chloroform	silica gel column chromatography	TLC	[24], [25]
**Milk**	Homogenization<br>Centrifugation<br>Filtration	NA[2]	IAC	UHPLC–ESI–MS/MS	[26]
**Milk**	NA[2]	Salt-induced liquid–liquid extraction (SI-LLE)	NA[2]	UHPLC–MS/MS	[27]
**Milk**	Centrifugation	Organic Solvents: acetonitrile<br>SPE columns	NA[2]	UHPLC–MS/MS	[28]
**Milk**	Centrifugation<br>Filtration	NA[2]	NA[2]	ELISA	[29], [30]
**Milk**	Centrifugation<br>Filtration	NA[2]	NA[2]	electrochemical immunosensor	[31]
**Milk**	Centrifugation<br>Filtration	NA[2]	NA[2]	Flexible Dispense-Printed Electrochemical Immunosensor	[32]
**Milk**	Homogenization	Organic Solvents: methanol	IAC	LC-FLD	[33]
**Cheese**	Centrifugation<br>Filtration	Organic Solvents: chloroform, hexane	IAC	HPLC-FLD	[17]
**Cheese**	Grated	Pepsin-HCl (P-HCl) method<br>Pepsin-pancreatin (PP) method<br>Cloroform (CH) method	IAC	HPLC-FLD	[2]

Table S4. (Continued).

Product	Sample Preparation	Extraction	Clean-up	Quantification Method	References
**Cheese**	Homogenization Centrifugation	C18-SPE	NA[2]	HPLC-FLD	[3]
**Cheese**	NI[1]	Organic Solvents: chloroform SPE	NA[2]	TLC	[34]
**Cheese**	Homogenization	Organic Solvents: dichloromethane/acetone<br>Organic Solvents: acetonitrile/water C18-SPE columns	NA[2]	UHPLC-MS/MS	[35]
**Cheese**	Homogenization	Organic Solvents: dichloromethane, methanol-water-hexane	IAC	HPLC-FLD, TLC, ELISA	[16]
**Cheese**	NI[1]	Organic Solvents: acetonitrile/water	IAC	HPLC-FLD	[15]
**Yoghurt**<br>**Cheese**	NI[1]	Organic solvents: dichloromethane, methanol, heptane	NA[2]	ELISA	[30], [36]
**Cheese**	NI[1]	Organic Solvents: chloroform, hexane	IAC	HPLC-FLD	[22]
**Cheese**	NI[1]	Organic solvents: dichloromethane	NA[2]	ELISA	[37]
**Cheese**	Homogenization	Organic Solvents: methanol	IAC	LC-FLD	[33]
**Yoghurt**	NI[1]	Organic Solvents: chloroform	IAC	HPLC-FLD, TLC, ELISA	[16]
**Yoghurt**<br>**Ice Cream**<br>**Cheese**	NI[1]	Organic Solvents: chloroform	silica gel column chromatography	TLC	[24]
**Yoghurt**	Homogenization	Organic Solvents: methanol	IAC	LC-FLD	[33]
**Butter**	NI[1]	Organic Solvents: chloroform, hexane	IAC	HPLC-FLD	[22]

NI [1] - Not Indicated; NA[2] - Not Applicable

References

[1] M. Hashemi, Z. Taherimaslak, and S. Rashidi, "Enhanced spectrofluorimetric determination of aflatoxin M1 in liquid milk after magnetic solid phase extraction," *Spectrochim. Acta - Part A Mol. Biomol. Spectrosc.*, vol. 128, pp. 583–590, 2014.

[2] A. Pietri, P. Fortunati, A. Mulazzi, and T. Bertuzzi, "Enzyme-assisted extraction for the HPLC determination of aflatoxin M1 in cheese," *Food Chem.*, vol. 192, pp. 235–241, 2016.

[3] A. C. Manetta *et al.*, "High-performance liquid chromatography with post-column derivatisation and fluorescence detection for sensitive determination of aflatoxin M1 in milk and cheese," *J. Chromatogr. A*, vol. 1083, no. 1–2, pp. 219–222, 2005.

[4] M. M. Vdovenko, C. C. Lu, F. Y. Yu, and I. Y. Sakharov, "Development of ultrasensitive direct chemiluminescent enzyme immunoassay for determination of aflatoxin M1 in milk," *Food Chem.*, vol. 158, pp. 310–314, 2014.

[5] L. Kanungo and S. Bhand, "Fluorimetric immunoassay for multianalysis of aflatoxins," *J. Anal. Methods Chem.*, vol. 2013, pp. 1–8, 2013.

[6] S. Rameil, P. Schubert, P. Grundmann, R. Dietrich, and E. Märtlbauer, "Use of 3-(4- hydroxyphenyl)propionic acid as electron donating compound in a potentiometric aflatoxin M1-immunosensor," *Anal. Chim. Acta*, vol. 661, no. 1, pp. 122–127, 2010.

[7] N. Michlig *et al.*, "Multiclass Compatible Sample Preparation for UHPLC–MS/MS Determination of Aflatoxin M1 in Raw Milk," *Chromatographia*, vol. 79, no. 17–18, pp. 1091–1100, Sep. 2016.

[8] J.-L. Liu, M. Zhao, Y. Zhuo, Y.-Q. Chai, and R. Yuan, "Highly Efficient Intramolecular Electrochemiluminescence Energy Transfer for Ultrasensitive Bioanalysis of Aflatoxin M1," *Chem. - A Eur. J.*, vol. 23, no. 8, pp. 1853–1859, Feb. 2017.

[9] G. Istamboulié, N. Paniel, L. Zara, L. R. Granados, L. Barthelmebs, and T. Noguer, "Development of an impedimetric aptasensor for the determination of aflatoxin M1 in milk," *Talanta*, vol. 146, pp. 464–469, 2016.

[10] S. M. Khoshfetrat, H. Bagheri, and M. A. Mehrgardi, "Visual electrochemiluminescence biosensing of aflatoxin M1 based on luminol-functionalized, silver nanoparticle-decorated graphene oxide," *Biosens. Bioelectron.*, vol. 100, pp. 382–388, Feb. 2018.

[11] L. C. Huang *et al.*, "Simultaneous determination of aflatoxin M1, ochratoxin A, zearalenone and α-zearalenol in milk by UHPLC-MS/MS," *Food Chem.*, vol. 146, pp. 242–249, 2014.

[12] N. S. Shuib, A. Makahleh, S. M. Salhimi, and B. Saad, "Determination of aflatoxin M 1 in milk and dairy products using high performance liquid chromatography-fluorescence with post column photochemical derivatization," *J. Chromatogr. A*, vol. 1510, pp. 51–56, 2017.

[13] M. Díaz-Bao, P. Regal, R. Barreiro, C. A. Fente, and A. Cepeda, "A facile method for the fabrication of magnetic molecularly imprinted stir-bars: A practical example with aflatoxins in baby foods," *J. Chromatogr. A*, vol. 1471, pp. 51–59, 2016.

[14] X. Guo, F. Wen, N. Zheng, and S. Li, "A qPCR aptasensor for sensitive detection of aflatoxin M1," *Anal. Bioanal. Chem.*, 2016.

[15] B. R. Yoon, S. Y. Hong, S. M. Cho, K. R. Lee, M. Kim, and S. H. Chung, "Aflatoxin M1 levels in dairy products from South Korea determined by high performance liquid chromatography with fluorescence detection," *J. Food Nutr. Res.*, vol. 55, no. 2, pp. 171–180, 2016.

[16] M. T. Al-Mossawei, L. A. Al-Zubaidi, I. S. Hamza, and S. Y. Abduljaleel, "Detection of AFM 1 in Milk and Some Dairy Products in Iraq using different techniques," *Advances in Life Science and Technology*, vol. 41, Online, pp. 74–81, 2016.

[17] A. M. Fernandes, B. Corrêa, R. E. Rosim, E. Kobashigawa, and C. A. F. Oliveira, "Distribution and stability of aflatoxin M1 during processing and storage of Minas Frescal cheese," *Food Control*, vol. 24, no. 1–2, pp. 104–108, Mar. 2012.

[18] M. M. Aguilera-Luiz, P. Plaza-Bolaños, R. Romero-González, J. L. M. Vidal, and A. G. Frenich, "Comparison of the efficiency of different extraction methods for the simultaneous determination of mycotoxins and pesticides in milk samples by ultra high-performance liquid chromatography-tandem mass spectrometry," *Anal. Bioanal. Chem.*, vol. 399, no. 8, pp. 2863–2875, Mar. 2011.

[19] S. Bodbodak, J. Hesari, S. H. Peighambardoust, and M. Mahkam, "Selective decontamination of aflatoxin M1 in milk by molecularly imprinted polymer coated on the surface of stainless steel plate," *Int. J. Dairy Technol.*, vol. 71, no. 4, pp. 868–878, 2018.

[20] Y. Rodríguez-Carrasco, L. Izzo, A. Gaspari, G. Graziani, J. Mañes, and A. Ritieni, "Simultaneous Determination of AFB1 and AFM1 in Milk Samples by Ultra High Performance Liquid Chromatography Coupled to Quadrupole Orbitrap Mass Spectrometry," *Beverages*, vol. 4, no. 2, p. 43, Jun. 2018.

[21] R. Norian, R. Mahmoudi, A. Porfarzaneh, F. Mashatian, and A. Kaboudari, "Determination of aflatoxin M 1 levels in raw milk samples using ELISA and high-performance liquid chromatography in Qazvin , Iran," *J. Mycol. Res.*, vol. 2, no. 1, pp. 41–48, 2015.

[22] S. Öztürk Yilmaz and A. Altinci, "Incidence of aflatoxin M1 contamination in milk, white cheese, kashar and butter from Sakarya, Turkey," *Food Sci. Technol.*, vol. 39, no. suppl 1, pp. 190–194, Jun. 2019.

[23] A. Curticapean and M. Curticapean, "Validation of new HPLC method determination of aflatoxin M1 in raw milk," *J. Environ. Prot. Ecol.*, vol. 14, no. 2, pp. 503–508, 2013.

[24] A. A. Fallah, "Aflatoxin M1 contamination in dairy products marketed in Iran during winter and summer," *Food Control*, vol. 21, no. 11, pp. 1478–1481, Nov. 2010.

[25] A. Kamkar, "A study on the occurrence of aflatoxin M1 in raw milk produced in Sarab city of Iran," *Food Control*, vol. 16, no. 7, pp. 593–599, Sep. 2005.

[26] A. Camaj, K. Meyer, B. Berisha, T. Arbneshi, and A. Haziri, "Aflatoxin M 1 contamination of raw cow's milk in five regions of Kosovo during 2016," *Mycotoxin Res.*, vol. 34, no. 3, pp. 205–209, Aug. 2018.

[27] L. Campone, A. L. Piccinelli, R. Celano, I. Pagano, M. Russo, and L. Rastrelli, "Rapid and automated analysis of aflatoxin M1 in milk and dairy products by online solid phase extraction coupled to ultra-high-pressure-liquid-chromatography tandem mass spectrometry," *J. Chromatogr. A*, vol. 1428, pp. 212–219, Jan. 2016.

[28] L. Campone *et al.*, "Occurrence of aflatoxin M1 in milk samples from Italy analysed by online-SPE UHPLC-MS/MS," *Nat. Prod. Res.*, vol. 32, no. 15, pp. 1803–1808, Aug. 2018.

[29] A. Mohamadi Sani, M. Khezri, and H. Moradnia, "Determination of Aflatoxin M1 in Milk by ELISA Technique in Mashad (Northeast of Iran)," *ISRN Toxicol.*, pp. 1–4, 2012.

[30] M. M. Motawee and D. J. McMahon, "Fate of aflatoxin M1 during manufacture and storage of feta cheese," *J. Food Sci.*, vol. 74, no. 5, pp. 42–45, 2009.

[31] L. Micheli, R. Grecco, M. Badea, D. Moscone, and G. Palleschi, "An electrochemical immunosensor for aflatoxin M1 determination in milk using screen-printed electrodes," *Biosens. Bioelectron.*, vol. 21, no. 4, pp. 588–596, Oct. 2005.

[32] B. D. Abera, A. Falco, P. Ibba, G. Cantarella, L. Petti, and P. Lugli, "Development of Flexible Dispense-Printed Electrochemical Immunosensor for Aflatoxin M1 Detection in Milk," *Sensors*, vol. 19, no. 18, pp. 1–11, Sep. 2019.

[33] M. H. Iha, C. B. Barbosa, I. A. Okada, and M. W. Trucksess, "Occurrence of aflatoxin M1 in dairy products in Brazil," *Food Control*, vol. 22, no. 12, pp. 1971–1974, 2011.

[34] A. Filazi, S. Ince, and F. Temamogullari, "Survey of the occurrence of aflatoxin M1 in cheeses produced by dairy ewe's milk in Urfa city, Turkey," *Ankara Univ. Vet. Fak. Derg.*, vol. 57, no. 3, pp. 197–199, 2010.

[35] B. Škrbić, I. Antić, and J. Živančev, "Presence of aflatoxin M1 in white and hard cheese samples from Serbia," *Food Control*, vol. 50, pp. 111–117, 2015.

[36] B. Sarimehmetoglu, O. Kuplulu, and T. Haluk Celik, "Detection of aflatoxin M1 in cheese samples by ELISA," *Food Control*, vol. 15, no. 1, pp. 45–49, Jan. 2004.

[37] H. R. Tavakoli, M. Riazipour, A. Kamkar, H. R. Shaldehi, and A. S. Mozaffari Nejad, "Occurrence of aflatoxin M1 in white cheese samples from Tehran, Iran," *Food Control*, vol. 23, no. 1, pp. 293–295, Jan. 2012.
